# Sodium Polystyrene Sulfonate-Induced Massive Bowel Necrosis With Distant Extraintestinal Crystal Deposition: A Case Report and Review of the Literature

**DOI:** 10.7759/cureus.71523

**Published:** 2024-10-15

**Authors:** Nebojsa Brezic, Ivana Milojevic, Ahmad Hassan, Katelyn Swanson, Tapan Bhavsar

**Affiliations:** 1 Department of Medicine, George Washington University School of Medicine and Health Sciences, Washington, DC, USA; 2 Department of Pathology and Laboratory Medicine, George Washington University School of Medicine and Health Sciences, Washington, DC, USA

**Keywords:** autopsy findings, bowel perforation, cation-exchange resin, drug-induced bowel injury, extraintestinal crystal deposition, sodium polystyrene sulfonate

## Abstract

Sodium polystyrene sulfonate (SPS), a cation-exchange resin, has been a mainstay in long-term hyperkalemia management but is associated with significant gastrointestinal complications, particularly when used with sorbitol. The deposition of SPS crystals within the intestinal mucosa has been suggested to precipitate ischemia, necrosis, and ulcerations, ultimately leading to bowel perforation. This case report details a striking instance of massive bowel perforation subsequent to SPS administration, with accompanying findings of disseminated crystals in distant organs and tissues upon autopsy. Additionally, we provide a comprehensive review of the existing literature on this rare yet significant drug-induced side effect.

## Introduction

Sodium polystyrene sulfonate (SPS), or Kayexalate, has been widely used for the long-term management of hyperkalemia from various causes since its approval by the FDA in 1958 [1]. This cation-exchange resin (CER), administered both orally and rectally, exchanges sodium for potassium in the colon, promoting potassium excretion into the stool. However, its effects are delayed, taking several hours to days to manifest, making it unsuitable for acute hyperkalemia management. SPS can also bind calcium ions in the intestinal lumen, potentially causing stool impaction and constipation, which are common side effects. To mitigate these effects, SPS was often administered alongside hypertonic sorbitol, a laxative that induces osmotic diarrhea [2]. The combination of SPS with sorbitol has been associated with serious complications like bowel necrosis and ulceration, leading to a black box warning for rectal use and the FDA's removal of 70% sorbitol-containing Kayexalate in 2006 [3]. Despite these measures, reports continue to link SPS without sorbitol to colonic ulceration, necrosis, and ischemia [4,5], suggesting that sorbitol may not be the sole culprit for these adverse events. The incidence of colonic necrosis after SPS administration ranges from 0.14% to 1.8% [1,3,6]. 

The first cases of SPS-associated colonic ulceration and necrosis were documented by Lillemoe et al., who reported five cases of colonic perforation linked to SPS in sorbitol, with four fatalities [7]. Initially, the cases involved SPS enema preparations, but there is now an established risk with both oral and rectal administration [8]. Upper gastrointestinal ulceration has also been reported; however, these cases have not required surgery due to a lower likelihood of progressing to transmural infarction, although some were fatal [2,9]. The pathophysiology of SPS-induced mucosal injury is yet to be fully understood. Initially, sorbitol was deemed responsible for ischemic enterocolitis, likely through mechanisms such as osmotic ischemia or prostaglandin effects [7]. Notably, 91% of patients with colonic perforation following SPS administration had a history of acute kidney injury, chronic kidney disease, or end-stage renal disease [1]. One suggested mechanism is the elevated renin in renal failure patients, leading to angiotensin II activation and splanchnic vasoconstriction, which predisposes the colonic mucosa to injury from electrolyte and fluid shifts [10]. Another plausible mechanism involves the local formation of neutrophil extracellular traps induced by crystals, reducing the metabolic activity of intestinal epithelial cells and triggering cell death and dysfunction of the epithelial barrier [11]. Risk factors for drug crystal-related intestinal necrosis include constipation, abnormal digestive secretion and absorption, intestinal hypoperfusion during hemodialysis, surgery, immunosuppressive or non-steroidal anti-inflammatory drugs, colonic distension, and chronic kidney disease [8].

## Case presentation

**Figure 1:**
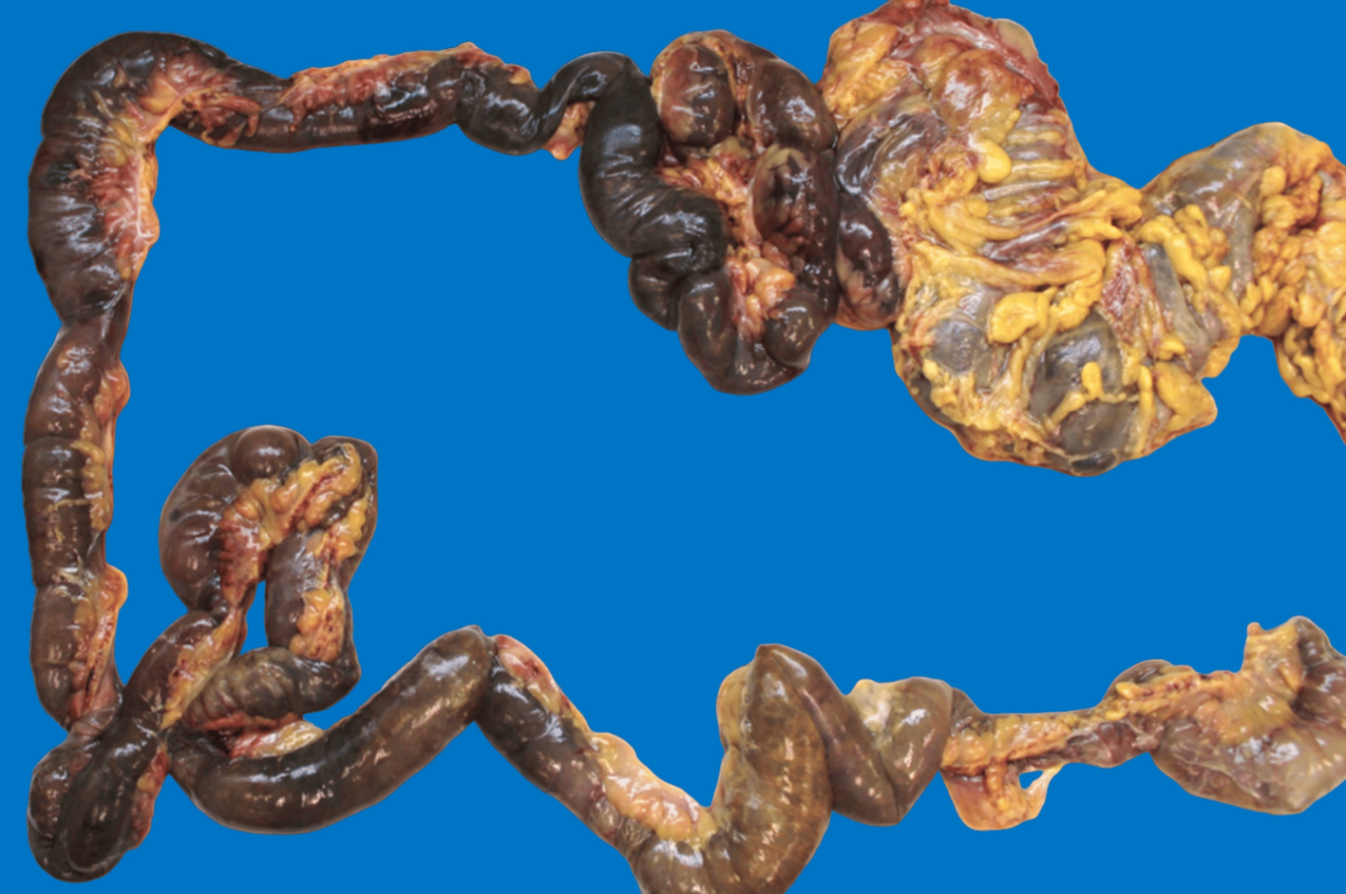
Extensive necrosis involving both the small and large bowel.

**Figure 2:**
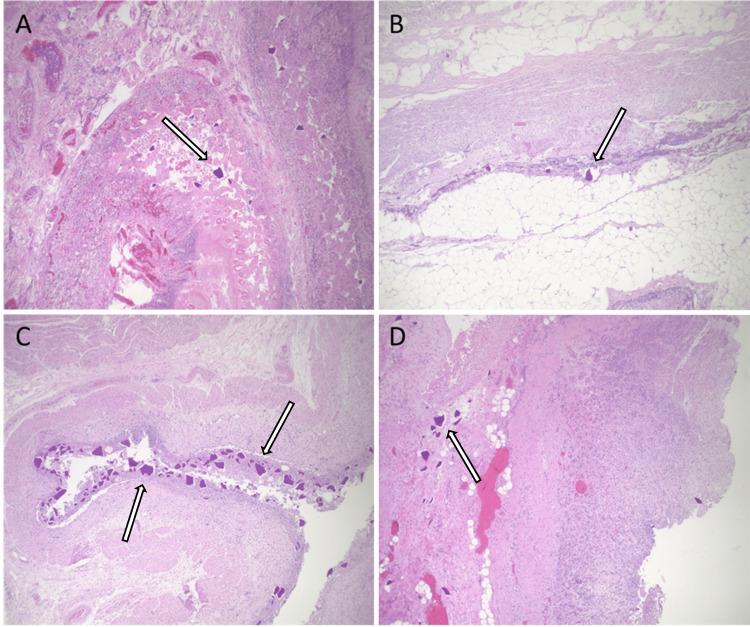
SPS crystal deposition in: small intestine mucosa with associated necrosis and fibrinopurulence at the site of perforation (A), large intestine with associated necrosis (B), gastroesophageal junction with associated fibrinopurulence (C) and muscularis of the stomach (D), H&E stain. SPS: sodium polystyrene sulfonate; H&E: hematoxylin and eosin

**Figure 3:**
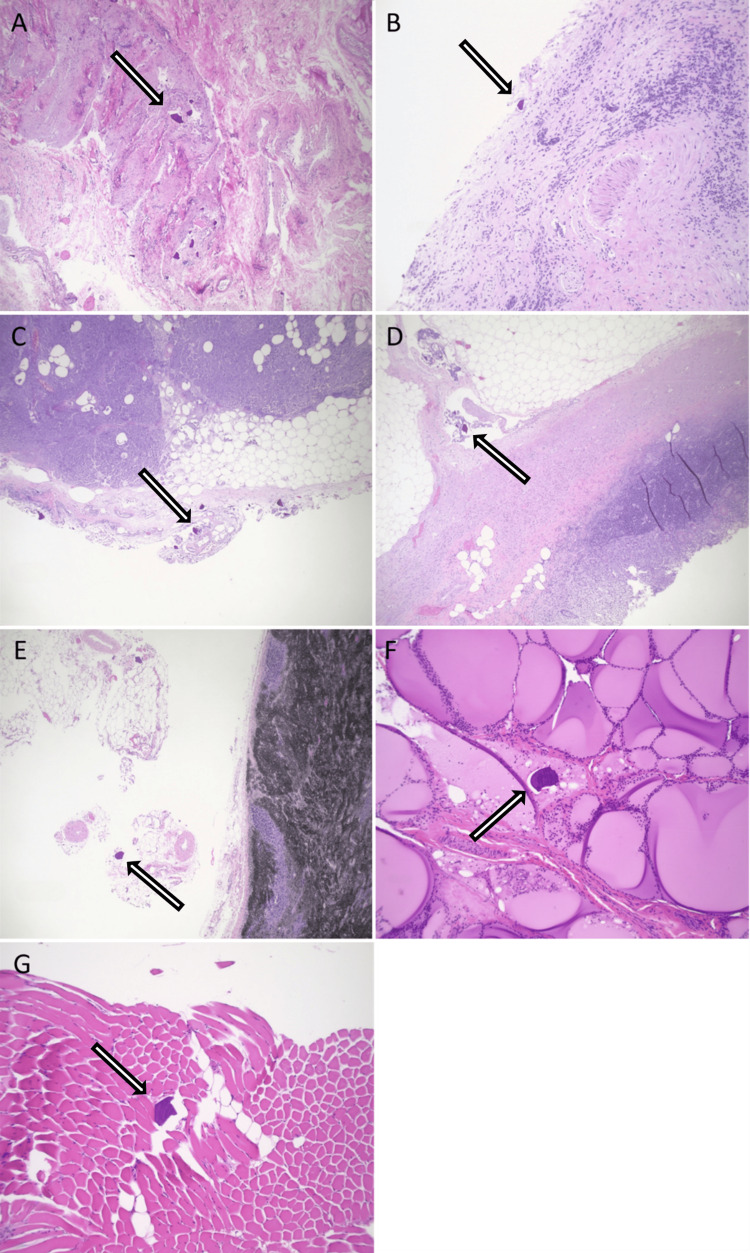
Systemic SPS crystal deposition in: (A) gallbladder mucosa, (B) urinary bladder, (C) peri-pancreatic adipose tissue, (D) peri-appendiceal adipose tissue, (E) peri-subcarinal lymph node adipose tissue, (F) thyroid gland follicles, and (G) skeletal muscle fibers, H&E stain. SPS: sodium polystyrene sulfonate; H&E: hematoxylin and eosin

**Figure 4:**
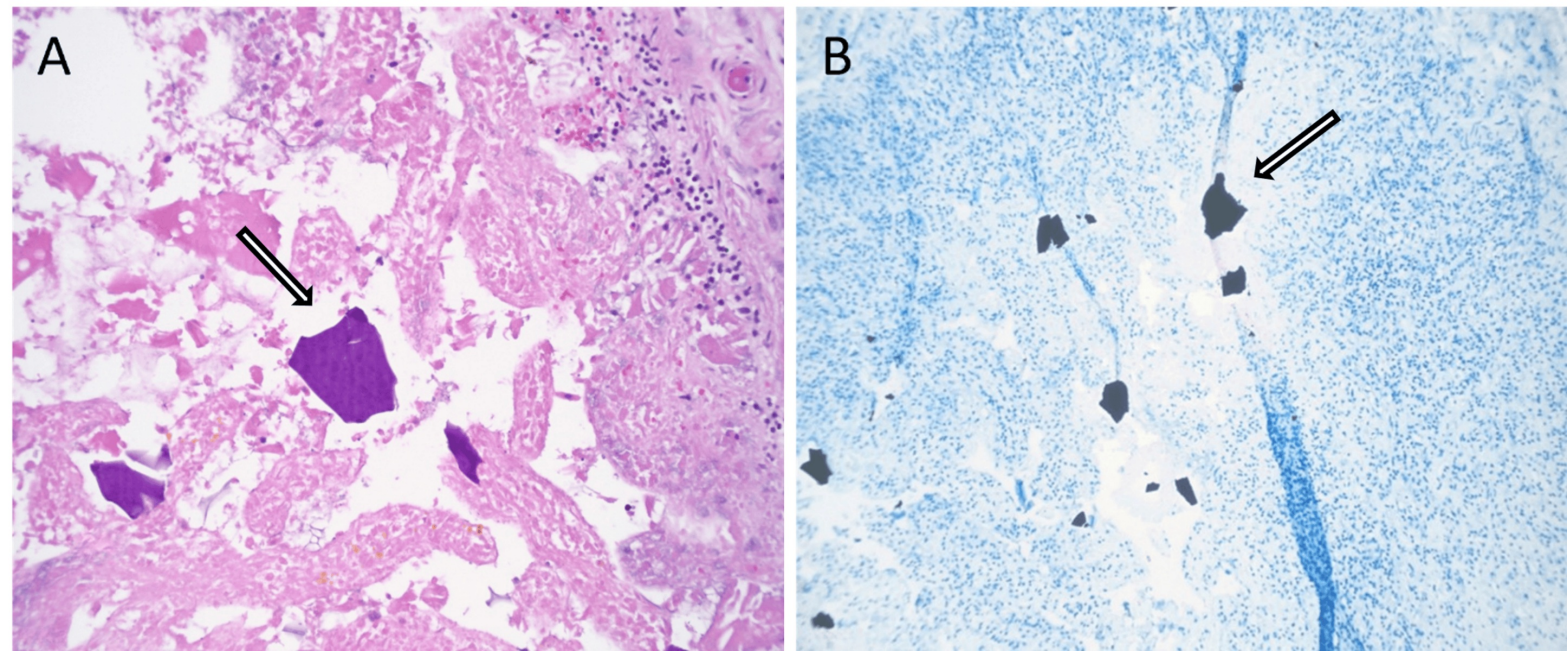
Typical "fish-scale" texture of SPS crystals on high magnification, H&E stain (A). AFB staining showcasing SPS crystals as black (B). SPS: sodium polystyrene sulfonate; H&E: hematoxylin and eosin; AFB: acid-fast bacillus

A 57-year-old African-American female with a complex past medical history of multiple fractures, traumatic brain injury, seizures, chronic respiratory failure requiring tracheostomy, ventilator dependence, and a gastrostomy tube secondary to polytrauma was transferred from a long-term acute care facility. She presented with worsening encephalopathy, acute kidney injury with hyperkalemia previously managed with SPS, and sepsis, suspected to be due to peritonitis. Upon arrival, the patient was hypotensive and tachypneic. Initial laboratory results indicated significant leukocytosis of 19,000/µL with a left shift and bandemia, an anion gap of 25 mEq/L, a potassium level of 7 mEq/L, a pH of 7.2, and a lactate level of 6.4 mmol/L. Physical examination revealed the patient to be alert but non-verbal, in moderate distress, and asynchronous with the ventilator, exhibiting signs of an acute abdomen. An exploratory laparotomy was performed, revealing diffuse gangrenous necrosis of the entire small and large intestines, extensive dense adhesions to the anterior abdominal wall, and an unidentified perforation with copious brown, foul-smelling fluid in the pelvis. An enterotomy occurred during abdominal entry, necessitating a segmental bowel resection. The skin was closed, and the bowels were left in discontinuity. The patient died 18 hours later after a decision was made not to escalate care further. Initial surgical pathology of the resected small bowel segment showed acute extensive mucosal necrosis with SPS crystals deposited in the mucosa. The final autopsy report revealed extensive necrosis of the small and large intestine (Figure 1) and widespread deposition of SPS crystals involving the gastroesophageal junction, stomach, small and large intestines, gallbladder, pancreas, spleen, adipose tissue surrounding the appendix, urinary bladder, thyroid gland, subcarinal lymph node, and skeletal muscles (Figures 2-3). Crystals were confirmed to be SPS by acid-fast bacillus (AFB) staining (Figure 4). No crystals were found in the lungs or brain. The foramen ovale of the heart was noted to be closed. The immediate cause of death was noted to be septic shock with multi-system organ failure, secondary to gangrenous necrosis of the bowel and peritonitis, attributed to SPS-induced gastrointestinal perforation.

## Discussion

SPS-induced necrosis can be distinguished from ischemic necrosis by the presence of SPS crystals in pathology specimens. These rhomboid or triangular, basophilic crystals, with a mosaic “fish-scale” pattern on hematoxylin and eosin (H&E) stain, are pathognomonic for SPS [12]. These crystals are typically found adhering to the mucosa or within an inflammatory milieu and ulceration. Hence, diagnosing SPS-induced bowel perforation necessitates identifying SPS crystals at sites of mucosal injury. However, other ion exchange resins, like sevelamer or cholestyramine, can morphologically resemble SPS crystals on conventional H&E stains. To discern between these crystals, AFB staining can be utilized: SPS crystals stain black, sevelamer crystals magenta, and cholestyramine crystals yellow [13,14]. This staining method offers a reliable means of differentiation. Additionally, SPS stains magenta in periodic acid-Schiff (PAS) and remains unstained in Congo red stain [6].

Reports of extraintestinal deposition of SPS crystals are infrequent. However, there have been documented cases of acute respiratory distress syndrome (ARDS) resulting from inadvertent inhalation during oral ingestion, with some cases even proving fatal [15]. There has also been a report of extraintestinal SPS in association with a perihepatic inflammatory pseudotumor developing adjacent to a colostomy site [16]. However, this is, to our knowledge, the first report of distant SPS crystal deposition outside of the alimentary tract. The systemic deposition of crystals, without right-to-left communication or persistent foramen ovale (PFO), remains a mystery. A case report on a giant intragastric SPS bezoar has proposed several mechanisms that could favor the precipitation of SPS, including the potential redissolution of SPS in the presence of high concentrations of multivalent salts [17]. However, these theories primarily stem from in vitro physical chemistry studies of SPS and may not fully reflect conditions within the body [18]. Yet, the answer might lie in the unique physicochemical properties of SPS, which could be influenced by factors such as gastric secretion and the local environment of the intestine. These factors may favor the depolymerization of SPS rather than precipitation, potentially allowing monomers to traverse bowel barriers and enter the systemic circulation, precipitating elsewhere, where the local milieu is more favorable. However, further studies are needed to thoroughly investigate these proposed mechanisms. Examining the chemical structure of extraintestinal SPS crystals could provide valuable insights into any deviations from their original form. Such analysis could help elucidate the underlying processes contributing to extraintestinal deposition and guide future research in this area. Nevertheless, the mechanism by which these crystals traverse from the gastrointestinal tract to distant sites remains unclear and poses an intriguing enigma. 

Reports of intestinal mucosal injury, including bowel perforation, have been documented with the use of Kalimate, or calcium polystyrene sulfonate (CPS), both with and without sorbitol, indicating a potential similarity in the mucosal injury mechanism to that observed with SPS [19]. The emergence of newer potassium-lowering agents, such as patiromer (Veltassa) and sodium zirconium cyclosilicate (Lokelma), presents clinicians with safer alternatives while maintaining efficacy in managing hyperkalemia. These medications not only effectively lower serum potassium levels but also demonstrate improved safety profiles compared to traditional options like SPS or CPS. With their enhanced tolerability and reduced risk of adverse effects, patiromer and sodium zirconium cyclosilicate offer valuable options for patients requiring long-term potassium management [20].

## Conclusions

Healthcare providers utilizing SPS for the management of hyperkalemia should maintain heightened awareness regarding the potential occurrence of life-threatening complications, particularly in patients presenting with additional risk factors. Despite its costliness, sodium zirconium cyclosilicate emerges as a potentially safer alternative, offering clinicians an avenue to mitigate the risks associated with traditional treatments while ensuring effective potassium management. Given the critical nature of hyperkalemia management and the evolving landscape of therapeutic options, a comprehensive evaluation of patient-specific factors, including risk profiles and financial considerations, is warranted to optimize treatment decisions and improve patient outcomes.
